# Comparing imputation approaches to handle systematically missing inputs in risk calculators

**DOI:** 10.1371/journal.pdig.0000712

**Published:** 2025-01-30

**Authors:** Anja Mühlemann, Philip Stange, Antoine Faul, Serena Lozza-Fiacco, Rowan Iskandar, Manuela Moraru, Susanne Theis, Petra Stute, Ben D. Spycher, David Ginsbourger

**Affiliations:** 1 Institute of Mathematical Statistics and Actuarial Science, University of Bern, Bern, Switzerland; 2 Gynäkopsychiatrie, Psychiatrie St. Gallen, St. Gallen, Switzerland; 3 Center for Evidence Synthesis in Health, Brown University, Providence, Rhode Island, United States of America; 4 Department of Obstetrics and Gynecology, University Women’s Hospital, Bern, Switzerland; 5 Department of Obstetrics and Gynecology, University Medical Center of the Johannes Gutenberg University Mainz, Mainz, Germany; 6 Institute of Social and Preventive Medicine, University of Bern, Bern, Switzerland; Stanford University School of Medicine, UNITED STATES OF AMERICA

## Abstract

Risk calculators based on statistical and/or mechanistic models have flourished and are increasingly available for a variety of diseases. However, in the day-to-day practice, their usage may be hampered by missing input variables. Certain measurements needed to calculate disease risk may be difficult to acquire, e.g. because they necessitate blood draws, and may be systematically missing in the population of interest. We compare several deterministic and probabilistic imputation approaches to surrogate predictions from risk calculators while accounting for uncertainty due to systematically missing inputs. The considered approaches predict missing inputs from available ones. In the case of probabilistic imputation, this leads to probabilistic prediction of the risk. We compare the methods using scoring techniques for forecast evaluation, with a focus on the Brier and CRPS scores. We also discuss the classification of patients into risk groups defined by thresholding predicted probabilities. While the considered procedures are not meant to replace fully-informed risk calculations, employing them to get first indications of risk distribution in the absence of at least one input parameter may find useful applications in medical practice. To illustrate this, we use the SCORE2 risk calculator for cardiovascular disease and a data set including medical data from 359 women, obtained from the gynecology department at the Inselspital in Bern, Switzerland. Using this data set, we mimic the situation where some input parameters, blood lipids and blood pressure, are systematically missing and compute the SCORE2 risk by probabilistic imputation of the missing variables based on the remaining input variables. We compare this approach to established imputation techniques like MICE by means of scoring rules and visualize in turn how probabilistic imputation can be used in sample size considerations.

## Author summary

Risk calculators and more generally, computer codes, play an important part in digital health. Given patient information, they allow for instance getting estimates for probabilities of developing certain diseases. Yet when part of the required patient information is missing, e.g., because some of the risk factors could not be measured, performing risk calculations may require to imputate missing values. We compare different imputation approaches, and essentially make a case that using probabilistic imputation approaches is worth the effort compared to deterministic approaches. In essence, propagating uncertainties on the imputated risk factors leads to probabilistic predictors of risks. We illustrate on the considered risks of developing a cardiovascular disease for cohort of patients from a menopause clinic in Bern, Switzerland, how the considered probabilistic approaches outperform deterministic ones in terms of forecast evaluation scores, and how such probabilistic risk predictions may be used in medical practice, highlighting in turn arising trade-offs between type I and type II errors.

## Introduction

Prediction is integral to the practice of medicine. Physicians need to infer a patient’s likelihood of developing a disease or prognosis when making decisions about the optimal course of action. To complement the traditional way of reasoning, statistical models for prediction have been increasingly used to estimate probabilities of (1) disease occurrences to guide testing decisions in a diagnostic workup and (2) outcomes of prognosis to inform therapeutic decision-making. A particular form of statistical prediction models, risk prediction models (hereinafter termed as *risk calculators*), is often employed to estimate the absolute risk of developing a disease within a pre-defined period of time (hereinafter termed as *risk score*). Risk scores can play a central role in communicating risk to individuals to motivate lifestyle changes and identifying individuals with elevated risks who will benefit from targeted prevention [1].

However, risk calculators are in general underutilized in clinical practice due to the time-consuming collection of all input variables. In principle, the development of a risk calculator requires data on the disease incidence based on a cohort of asymptomatic individuals, established associations between known risk factors and incidence, and a statistical model that quantifies the relationship between the incidence and risk factors. Ideally, we know all necessary inputs of a risk calculator in order to better discriminate between those with a high vs. low risk of developing the disease. However, it may happen for some cases that part of these risk factors needed as input to the risk calculator cannot be readily collected due to time constraints or cost considerations. For example, the European Systematic Coronary Risk Evaluation 2 (SCORE2) [2] algorithm developed to estimate the risk of cardiovascular disease (CVD) in Europe requires information on age, blood lipid levels (HDL and total cholesterol), systolic blood pressure, and personal history of diabetes mellitus. Additionally, there is a parameter for the “risk region”, which we have set to “low-risk” for Switzerland (see Figure 2 in [2]) throughout this work. Gathering part of the information necessitates multiple visits to outpatient clinics for sample collection, laboratory work, and doctor consultations. In some cases, part of these inputs may be lacking. Missing data is a common and important issue in empirical studies, having led to a number of developments. Reasons for data to be missing may vary depending on the context. One typically differentiates between *systematically missing data* versus *occasionally missing data*. In the present context, we are primarily focusing on systematically missing data, meaning that we wish to estimate risks when some prescribed inputs are systematically lacking.

In broader missing data settings, the most obvious approach to missing data consists in omitting columns (patients) for whom some values are missing [3], which would be completely counterproductive in the considered situation. Another approach that is often used due to its simplicity to deal with missing information on some risk factors is called patching, and consists in plugging in the mean (which can be generalized to any reference value based on the input’s distribution) of any missing variable in place of the missing values [4].

More generally, several imputation approaches have been used to fill in missing values in deterministic or probabilistic ways. Depending on the reasons for missing data to occur, the imputation technique may introduce biases. Using probabilistic imputation, one aims to better reflect the fact that the missing variables are unknown. Some of these methods have already been compared on medical data sets, when specifying a structure for the missingness mechanism [5]. In our application, we use our knowledge of other patients to probabilistically impute a certain patient’s clinical parameters.

The goal of this paper is to investigate and compare competing imputation methods for estimating risk scores in situations where information on certain risk factors is missing. We demonstrate the considered approaches, as well as our comparison approach using scoring rules from the realm of probabilistic forecast, in a context of cardiovascular risk calculation with SCORE2, used and surrogated for a cohort of patients from a menopause clinic in Bern, Switzerland. Let us remark that while our leading example is from a non-communicable disease context, the presented work is generic and could be applied to a variety of situations, notably to risk assessment in the context of infectious diseases. In the broad field of predictive medicine, the use of risk calculators to evaluate the probability (under a prescribed model) for a patient to develop a disease in the future has in fact become quite common. In the specific cardiovascular case study considered throughout the paper, the output (or *risk score*, which echos with another notion of score employed in the following in the context of probabilistic forecast evaluation) stands for a probability of developing a cardio-vascular disease in the next 10 years. Such probabilities may be of potential use, for instance, when conducting probabilistic recommendations for moderate versus high risk patients.

The considered SCORE2 risk calculator relies on a logistic regression model fitted based on physiological and clinical measurements, and delivers a probability that an event takes place given some patient specific inputs. More specifically, the model was trained by the European Society for Cardiology (ESC) in 2021 on labeled data sets for different regions depending on their “risk level”. The fitted parameters are then used to calculate the risk of a patient as a function of the inputs.

Let us stress however that the imputation approaches considered here do not at all require a risk calculator based on logistic regression, and actually tailored approaches might be better adapted for the latter case. In contrast, the SCORE2 risk is merely used here for illustration purposes and could be replaced by other types of simulators. We consider in fact the risk calculator to be an unknown “black box”, since not all risk calculators reveal detailed information about the underlying input-output relationship, and our goal is to cover general settings without specific statistical and/or mechanistic model assumptions. In the approaches we present in this paper we tackle the situation where some input parameters of such risk calculators are missing, so that uncertainties are propagated to the simulator’s output and the output risk is hence being estimated rather than merely calculated.

An important point is that, if we specify point estimates for a patient’s missing inputs, the resulting point estimate of the risk gives no information about the associated uncertainty. As an alternative we tend to advocate a probabilistic approach with a propagation on uncertainties beyond point estimates. While such a probabilistic approach may improve decision-making, as our results illustrate, it appears to not be broadly used in the field of medical risk calculations yet.

Probabilistic prediction is an important tool in science and society (e.g. weather forecast, economics) and was recently increasingly used is health sciences to predict infectious diseases outbreaks in certain populations [6–10]. In this work, we compare five imputation approaches, coined I1 to I5, where I1, I2 and I3 provide point predictions while I4 and I5 provide probabilistic ones. I1 consists in replacing missing inputs by their (unconditional) means. I2 is a pooled variant of Multivariate Imputation with Chain-Equation (MICE). In I3 and I4, an approach is used to approximate the joint distribution of inputs and conditioning is used for imputation. I3 simply uses conditional expectations as plug-in estimates, while I4 propagates the conditional distribution of unknown inputs given known ones to the resulting risk. I5 is a probabilistic extension of the MICE approach I2.

In order to compare the performances of these approaches at capturing the actual risks, we appeal to scoring approaches from the world of forecast evaluation. While point predictions are usually evaluated by means of loss or cost functions, that is a mathematical function that measures the difference between the prediction and the actual observed outcome, another tool is necessary to evaluate probabilistic predictions, namely *scoring rules*. This requirement derives from the fact that for probabilistic predictions, predictive distributions must be compared to the observed outcome. Following [11], probabilistic predictions should maximize the sharpness of the predictive distributions subject to calibration. Although other evaluation tools exist, proper scoring rules have been established in the last years as the most popular evaluation method in this context and used whenever probabilistic forecasts are involved. The reason for this is that proper scoring rules address calibration as well as sharpness and therefore allow us to compare and rank different prediction methods. The Continuously Ranked Probability Score (CRPS) [11] and Brier score [12, 13] are considered here, the latter being focusing specifically on the ability of classification [14, 15]. In clinical applications this has lately become an important tool when it comes to quantifying/comparing probabilistic predictions/methods [16, 17], with a special interest in comparing tools to classify whether or not a patient falls in a prescribed “high risk” category [18–20]. Throughout the paper, we consider leave-one-out settings and compare (via scoring) for every left-out patient their actual risk with the (deterministic or probabilistic) predictions delivered by the imputation methods. Our experimental results highlight the added value of probabilistic imputation, with superior results for I4 and I5 in terms of CRPS and Brier scores. Also, coming to the down-to-earth problem of performing a pre-triaging of high-risk patients from probabilistic classifications, we numerically investigate the effect of varying the cut-off in probability thresholding on the trade-off between induced type I and type II errors.

The paper is structured as follows. In Section Methodology, we focus on a more detailed presentation of the methodology, from the five considered imputation approaches to the evaluation of the resulting risk predictions. Section Implementation and results is dedicated to implementation and results, starting with a presentation of the data and its exploratory analysis, followed by implementation details concerning the imputation approaches, some didactic examples highlighting how the considered workflow works with a selection of artificial patients, and the actual benchmark of I1-I2 in terms of CRPS and Brier scores. Additional discussion points are tackled in Section Discussion, including the sensitivity of I4 and I5 to the cut-off level when thresholding the resulting probabilities, an opening to broader distribution classes and inference approaches for probabilistic imputation via joint distribution modelling, and remarks and perspectives regarding the use of extra covariates in the considered context. Section Conclusion concludes the paper.

## Methodology

### Probabilistic imputation of missing risk calculator parameters

**Fig 1:**
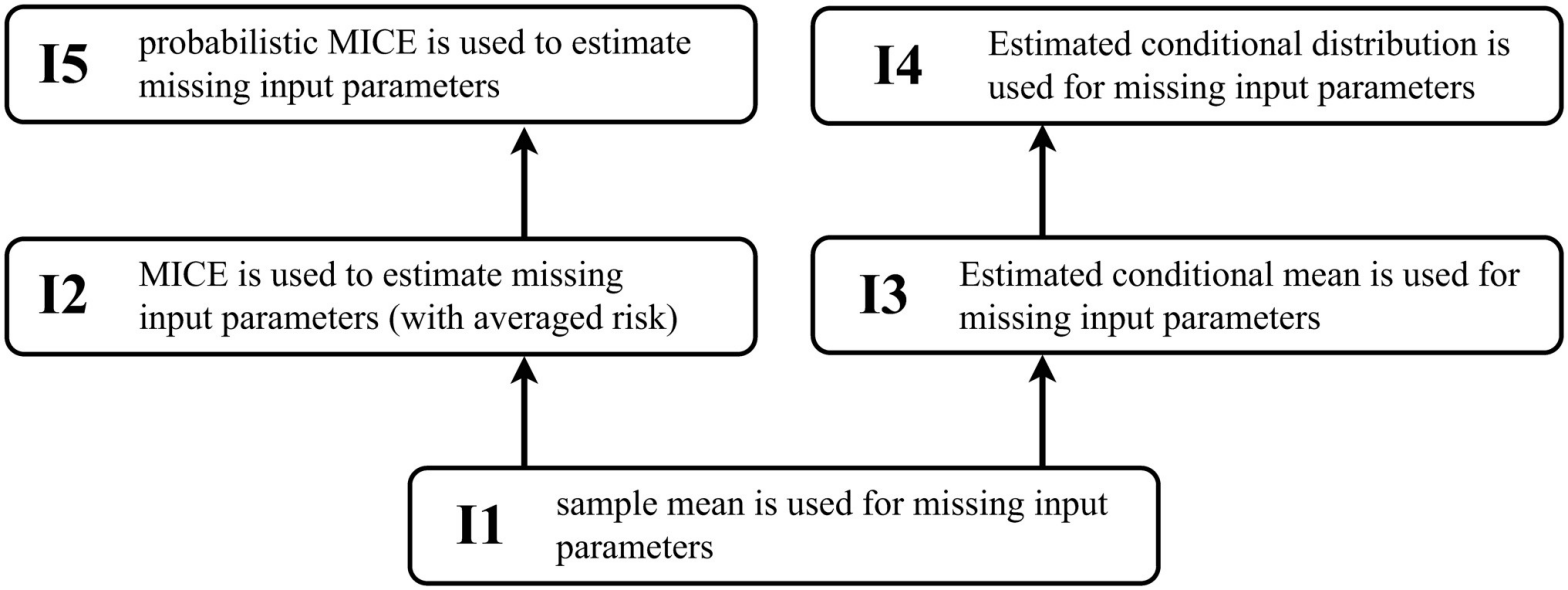
The above figure provides a rough summary of the five methods we examine with respect to their quality in predicting systematically missing values. Method I1 is a naive approach to dealing with missing values. Methods I2 and I3 provide more elaborate approaches. Methods I4 and I5 extend method I3 and I2, respectively, by providing probabilistic predictions.

This section provides an overview of the methods we implemented here to deal with systematically missing values, as summarized by Fig 1.

The covariates necessary for calculating the SCORE2 risk (apart from smoking behavior and the incidence of diabetes mellitus) are age, systolic blood pressure (BP), total cholesterol (TotalC), and HDL cholesterol (HDLC). We refer to BP, TotalC, and HDLC as the *unknown variables* due to the need for medical examinations to determine these values, whereas age can clearly be considered a *known variable*.

The first imputation approach we consider here, coined I1, consists in evaluating the risk calculator of interest with known inputs (age, smoking habit and diabetes) set to their actual respective values and substitute the unknown inputs (TotalC, HDLC and BP) by the sample mean of those values.

Another considered approach to deal with missing values is the so-called multiple imputation by chained equations (MICE) [21] method. This method is widely used in many fields, and particularly in medicine, due to its simplicity and capacity to handle many different data types. The idea of multiple imputation is to generate several plausible imputed data-sets and to combine them into one guess in order to reduce the uncertainty of the imputation. When missing values are present in different variables, MICE provides a sequential multivariate procedure for imputation. This algorithm is also called *fully conditional specification* and is very similar to Gibbs sampling. The first step is to fill in missing values with random draws from observed data. Next the missing values are imputed on a variable by variable basis. In each iteration it runs a regression of one of the missing values and draws from the distribution of the fitted model to obtain the new input. The process is iterated over all variables with missing values, which correspond to one iteration of the algorithm. The number of iterations is chosen in order to meet some convergence criterion. This gives us one imputed data-set and this methodology is applied several times in parallel to get multiple imputed data-sets. Subsequently, we applied the SCORE2 calculator to every imputed data-set and we used, for each patient, the mean of the predicted risks to get a single point estimate. In our implementation, we used predictive mean matching as regression procedure inside the MICE algorithm and we chose the number of multiple imputations (parameter *m* in the R-package [22]) via cross-validation. This approach was denoted I2. However, taking the mean in order to come up with a point estimate defeats one of the main purposes of multiple imputation, namely to propagate the uncertainty of the missing values. In typical applications this uncertainty is propagated to the model parameters being estimated (by Rubin’s rules for the variance) and in ours it should be propagated to predicted risk by simply taking the full distribution. The result is a probabilistic prediction based on MICE and denoted by I5.

The two remaining approaches I3 and I4 rely on directly modelling the joint distribution of inputs. In I3, we revisit I1 but instead of plugging-in the unconditional means of the missing input parameters, we plug in the conditional means under a joint statistical model for the vector of all input parameters. While this could tentatively be used with further copula models to learn this joint distribution, we proceed here for simplicity with an approach where the marginal distributions are first learnt, and then empirical Gaussian conditioning is performed after marginal transformations, followed by back transformations.

A similar workflow is applied in I4, except that instead of plugging-in conditional expectations for the missing inputs, one propagates their obtained conditional distributions and obtain a conditional distribution for the risk.

It is possible, and as we will see (Section Extra covariates), also advantageous, to introduce *extra variables* for all approaches except I1, where adding more information does not have any impact. Extra variables are simply variables that are not directly needed for the calculation of the SCORE2 risk but are easily accessible to a patient and can be considered as additional knowledge. Therefore, we will include the patient’s BMI in our set of *known variables* and tacitly rely on it when performing approaches I2-I5.

Ins and outs of implementing these approaches are further discussed in Section Implementation and results.

### Evaluation of the resulting risk distribution

One of our main goals in this work is to evaluate the five different approaches introduced above, and to find out how they compare at predicting a patient’s SCORE2 risk. Classically, predictions are evaluated by means of scoring functions in the case of point predictions, and scoring rules in the case of probabilistic predictions; see [23] and [11]. Methods I1, I2 and I3 yield point predictions, while I4 and I5 yield probabilistic predictions. To fairly compare their performance, however, the same scoring rule should be used. To this end, we interpret our point forecasts as probabilistic forecasts in the sense of Dirac distributions (probability distributions concentrated on one point).

The first scoring rule we consider here is the Continuous Ranked Probability Score (CRPS). This scoring rule is widely used as a measure of performance in the case of available cumulative distribution function (CDF) of the probabilistic forecast. The CRPS is defined to be the integrated squared deviation between the forecast’s cumulative distribution function *F* and the empirical cumulative distribution function of the observation *y* that has materialized, i.e.

$$\begin{array}{c} \text{CRPS}\left(F,y\right)=\int_{R}{\left(F\left(x\right)-1\left\{x\geq y\right\}\right)}^{2}\text{d}x, \end{array}$$

where **1** denotes the indicator function [24]. The CRPS generalizes the Mean Squared Error to probabilistic forecasts, which is one of the reasons why the CRPS is one of the most widely used accuracy metrics when probabilistic forecasts are at hand.

Since our imputation approach relies on Monte-Carlo simulations we only know the empirical CDF of our forecast. To this end we estimate the CRPS by replacing the true CDF *F* by the empirical CDF $\hat{F}$

$$\begin{array}{c} \text{CRPS}\left(\hat{F},y\right)=\int_{R}{\left(\hat{F}\left(x\right)-1\left\{x\geq y\right\}\right)}^{2}\text{d}x. \end{array}$$

Over all patients *i*, the average CRPS for the methods is therefore given by

$$\begin{array}{c} \bar{\text{CRPS}}=\frac{1}{n}\sum_{i=1}^{n}\text{CRPS}\left({\hat{F}}_{i},y_{i}\right), \end{array}$$

where ${\hat{F}}_{i}$ is based on all patients but the *i*th. For forecasts with finite first moment, the CRPS can equivalently be written in terms of expectations [11]. Based on this alternative definition, other estimates for the CRPS are also available. In this paper, however, we focus on the estimate based on the classical integral definition. The reason for the broad use of the CRPS is at least partly due to the fact that the CRPS is a proper scoring rule, meaning that the true forecast distribution does indeed minimize the expected CRPS.

In practice, predicted risks are often classified into risk levels e.g. moderate versus high. We therefore additionally considered the Brier score for evaluation [12]. The Brier score allows us to examine the accuracy of a probabilistic binary forecast. Additionally, it is closely related to the mean squared error, as in both methods the quadratic difference between the outcome and the probability of the outcome are compared. For example, if we define a patient to be low risk if their SCORE2 risk is below 1%, then the outcome *y* of a low risk patient is 1, otherwise it is 0. Moreover, the probability of a patient to be low risk is $\hat{F}\left(0.01\right)$. The Brier score of a patient is therefore given by

$$\begin{array}{c} {\text{Brier}}_{<1\%}\left(\hat{F},y\right)={\left(\hat{F}\left(0.01\right)-y\right)}^{2}. \end{array}$$

Across all patients, the average Brier score for a risk below 1% is therefore given by

$$\begin{array}{c} {\bar{\text{Brier}}}_{<1\%}=\frac{1}{n}\sum_{i=1}^{n}{\left(\hat{F_{i}}\left(0.01\right)-y_{i}\right)}^{2}, \end{array}$$

where the *y*_*i*_’s are 0 if the calculated risk of the *i*th patient is < 1%.

In addition to the forecasts ability to differentiate between risks below and above 1%, we also want to evaluate the forecasts ability to predict a risk above 5%. With the same reasoning as before the average Brier score is then

$$\begin{array}{c} {\bar{\text{Brier}}}_{>5\%}=\frac{1}{n}\sum_{i=1}^{n}{\left(1-\hat{F_{i}}\left(0.05\right)-y_{i}\right)}^{2}, \end{array}$$

where the *y*_*i*_’s are 0 if the calculated risk of the *i*th patient is > 5%.

### Ethical statement

Health-related personal data was collected in the Cimbolic study (Project-ID 2016-01179) to perform a retrospective study. Documented refusal was not available in any of the patient files (as per clinical trial protocol approved by the cantonal ethics committee No 2016-01179). Parts of this dataset are reused in the present project.

The ethics committee of canton Bern declared on 10th January 2023 that the focus of the current study is on statistical methods and not affected by the requirements of the law on human research (Project-ID 2022-02258). Usage and publication of the anonymized data has been approved by the ethics committee of the Kanton of Bern (Req-2024-00947).

## Implementation and results

### Exploratory data analysis

The considered data set was collected at the gynecology department of the university hospital (Inselspital) in Bern, Switzerland, and contains the age, smoking habit, systolic blood pressure (BP), total cholesterol (TotalC), HDL cholesterol (HDLC), incidence of diabetes (Diabetes) and the body mass index (BMI) of 359 female patients aged between 40 and 69 years. The data was collected in the context of [25].

To illustrate our approach, we use the SCORE2 risk calculator to estimate 10-year fatal and non-fatal cardiovascular disease risk in individuals without previous cardiovascular disease or diabetes aged 40-69 years, based on a European reference group [2].

In a first step, the data was analyzed descriptively. To this end histograms and boxplots were drawn for the continuous variables and frequencies were calculated for the categorical variables.

Of the 359 female patients there were 285 non-smokers and 74 smokers. None of the women was treated for blood pressure.

**Fig 2:**
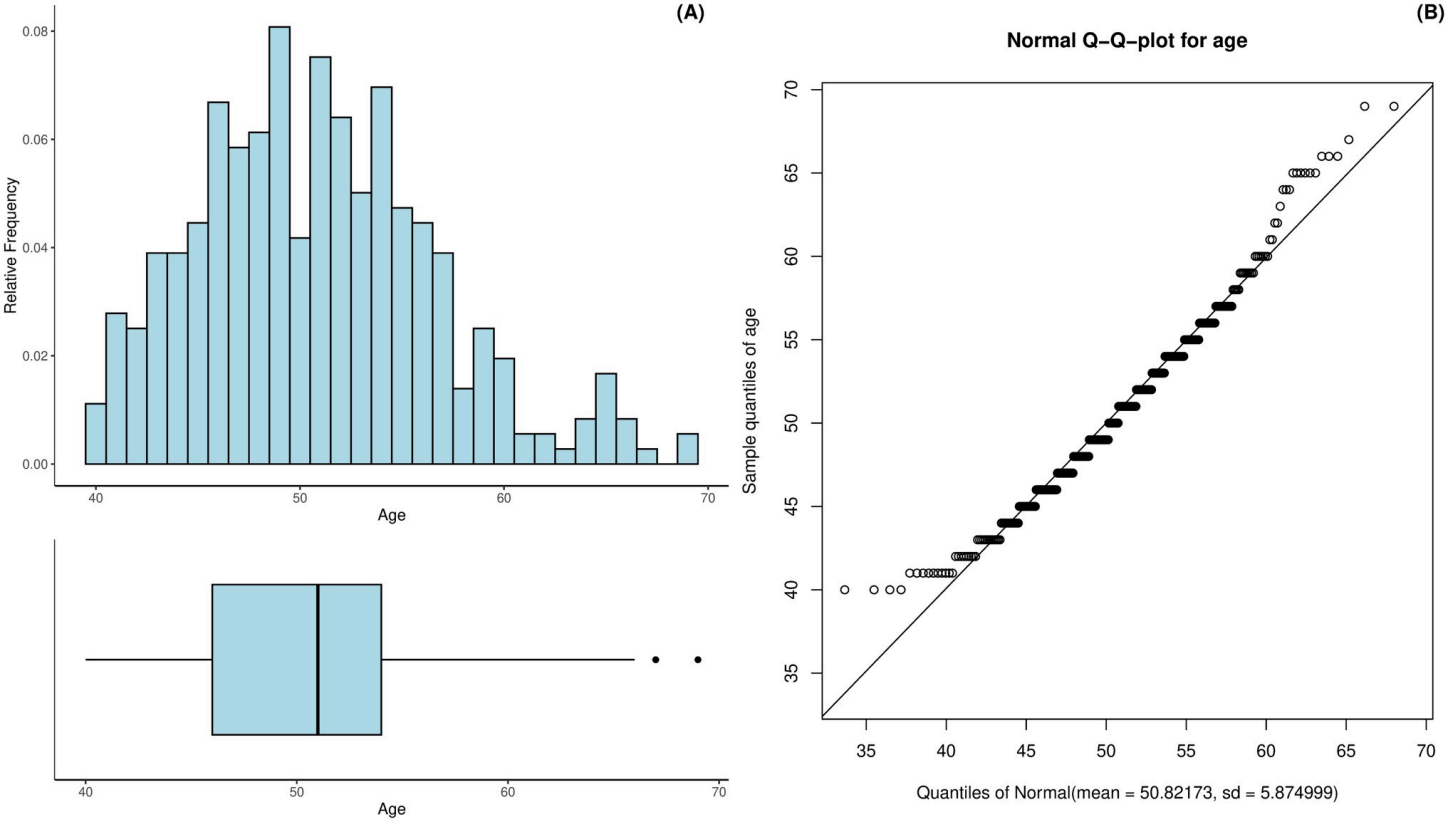
Histogram and boxplot of the patients’ age (A) as well as the Normal Q-Q-plot for the estimated age distribution (B).

The age of the patients ranged from 40 to 69 years of age with a mean age of 50.82 and a standard deviation of 5.67 years. A proportion of 50% of women were between 46 and 54 years old. Fig 2 shows that the ages were roughly symmetrically distributed with a skew of 0.52. Moreover, the Q-Q-plot in Fig 2 suggests that the age distribution can be approximated reasonably well by a Gaussian distribution (for instrumental purpose, by remaining aware of the unsuitable real support). The excess kurtosis of the age was 0.09.

**Fig 3:**
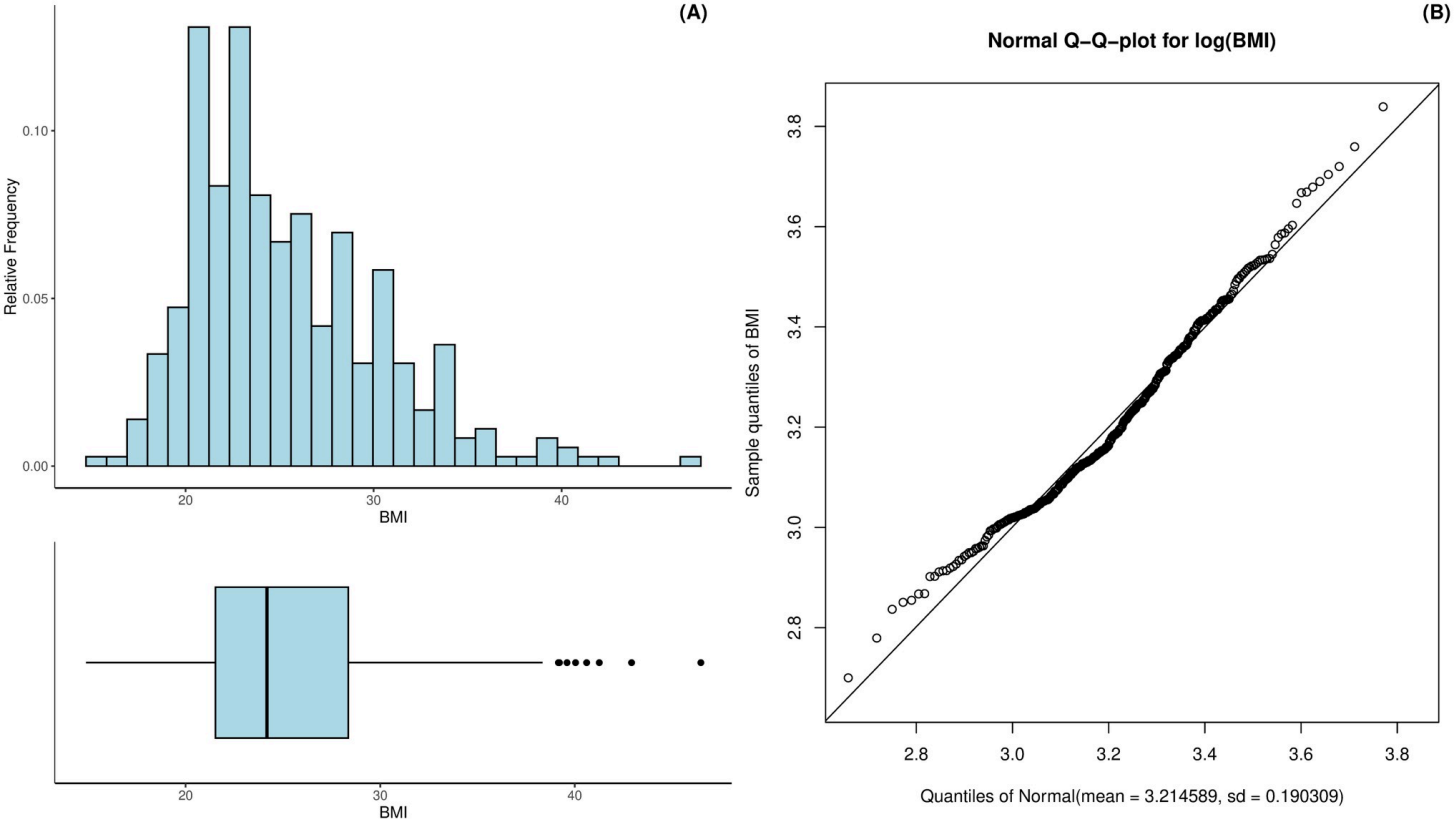
Histogram and boxplot of the patients’ BMI (A) as well as the log-Normal Q-Q-plot for the estimated BMI distribution (B).

The BMI of the patients ranges from 14.88 to 46.48 whereas half of the patients have a BMI between 21.53 and 28.36 kg/m^2^; see Fig 3. The mean BMI was 25.36 with a standard deviation of 5.06. The distribution is left skewed (skew equals 0.95) and can best be approximated via a log-normal distribution as Fig 3 shows. The excess kurtosis of the BMI is 0.96.

**Fig 4:**
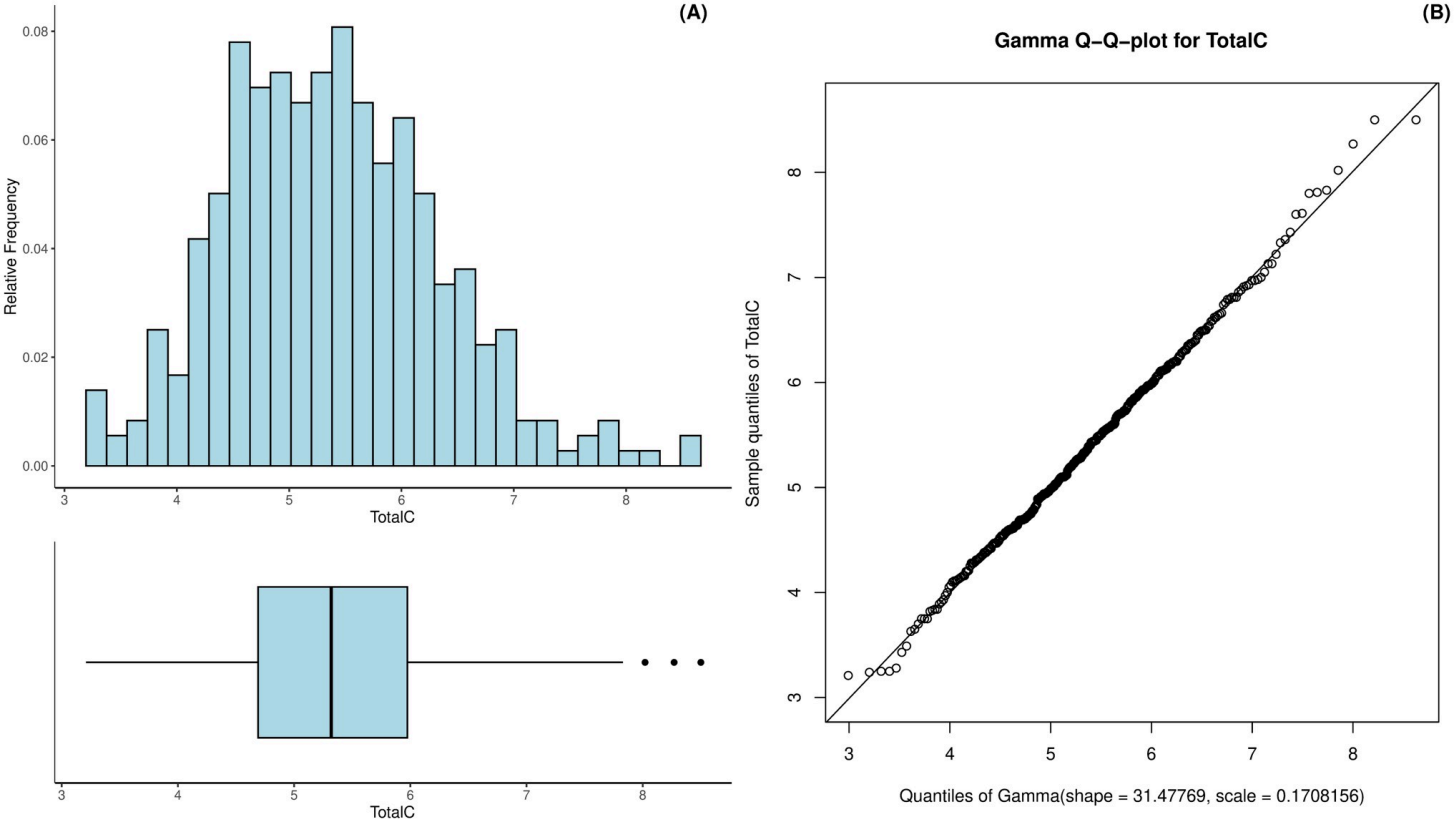
Histogram and boxplot of the patients’ total cholesterol (A) as well as the Gamma Q-Q-plot for the estimated total cholesterol distribution (B).

The total cholesterol in the patients was measured in mmol/L and ranged between 3.21 mmol/L to 8.5 mmol/L with a mean of 5.38 and a standard deviation of 0.96 mmol/L; see Fig 4. The distribution is slightly left-skewed (skew equals 0.40) and can be approximated via a gamma distribution as shown in Fig 4. The excess kurtosis of the total cholesterol was 0.23.

**Fig 5:**
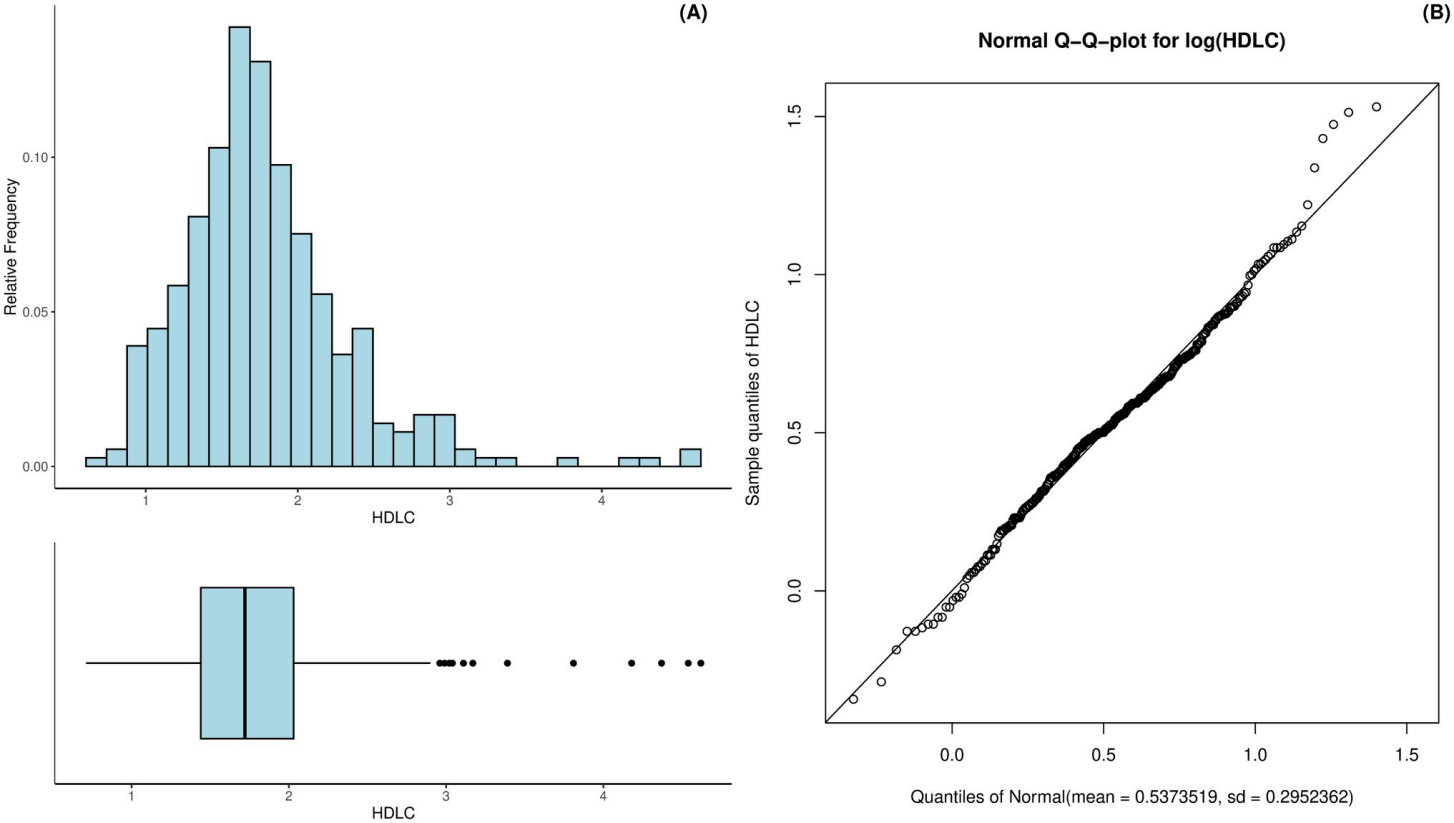
Histogram and boxplot of the patients’ hdl cholesterol (A) as well as the log-Normal Q-Q-plot for the estimated hdl cholesterol distribution (B).

The HDL cholesterol in the patients was also measured in mmol/L and ranged between 0.71 mmol/L to 4.62 mmol/L with a mean of 1.79 and a standard deviation of 0.56 mmol/L; see Fig 5. The distribution is again slightly left-skewed with a skew of 1.52 and is approximated as well by a log-normal distribution as shown in Fig 5. However, the tails seem to be heavier than we would expect in a log-normal distribution. The excess kurtosis of the HDL cholesterol is 4.67.

**Fig 6:**
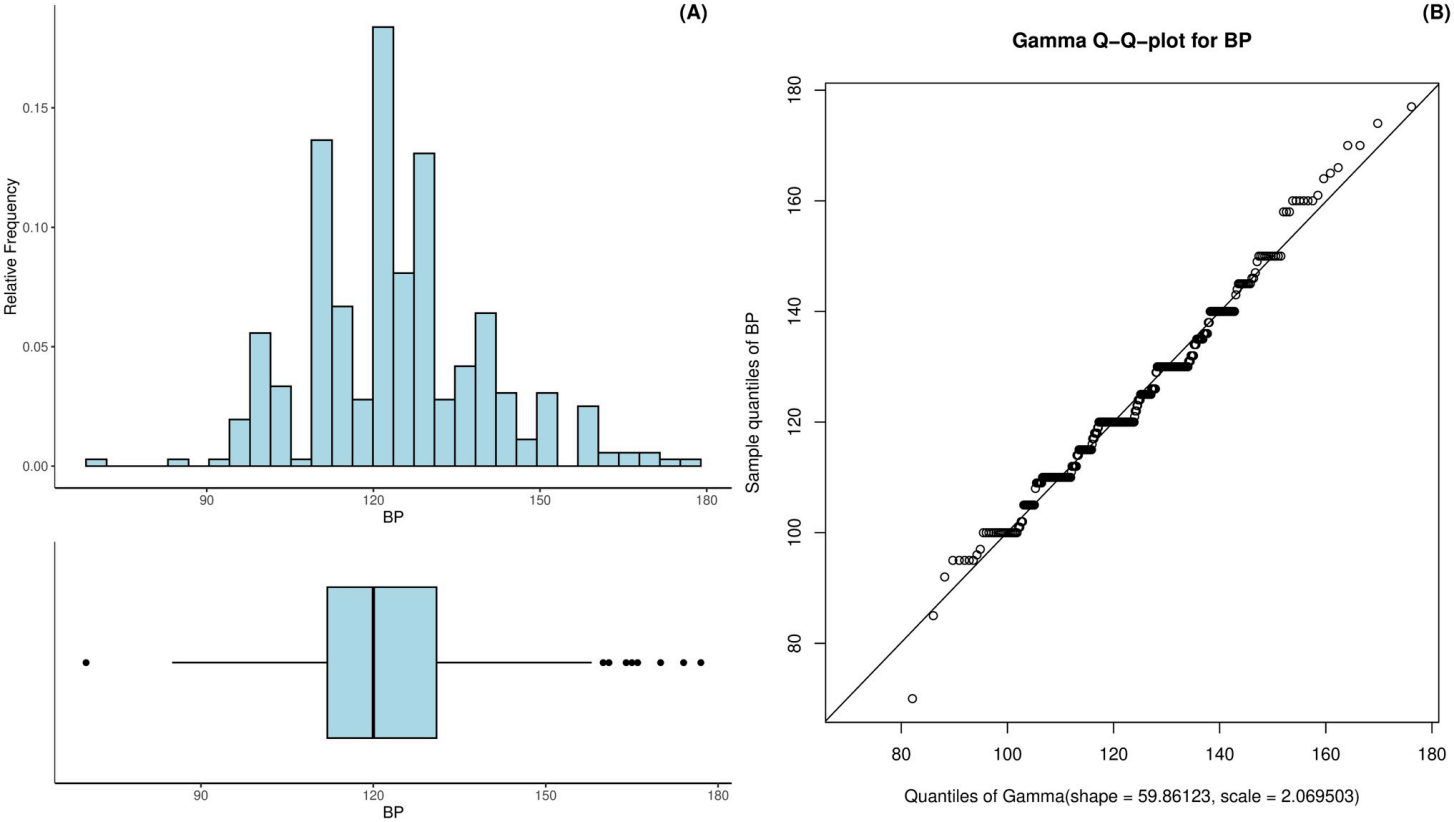
Histogram and boxplot of the patients’ systolic blood pressure (A) as well as the Gamma Q-Q-plot for the estimated systolic blood pressure distribution (B).

The systolic blood pressure in the patients ranged from 70 to 177 mmHg with a mean of 123.88 mmHg and a standard deviation of 16.03. Half of the patients had a blood pressure between 112 and 131 mmHg. Fig 6 gives more information on the observed values. The histogram suggests that there is some rounding present in the data. The Q-Q-plot in Fig 6 suggests the fitted gamma distribution to deliver a reasonable approximation for the systolic blood pressure distribution. The skew of the systolic blood pressure equals 0.47 and the excess kurtosis equals 0.58.

### Implementation of I1, I2, I3, I4 and I5

In this subsection we focus on the implementation of the approaches that are introduced in Section Probabilistic imputation of missing risk calculator parameters. For the basic approach I1 we just have to calculate the sample means of BP, TotalC and HDLC from the underlying data set, which is straightforward. We can then use the known inputs age, smoking habit and diabetes of a single patient and substitute the unknown inputs (BP, TotalC, HDLC) with the corresponding sample mean.

For the implementations of the MICE approaches I2 and I5, we have used the available R-package mice[22]. For a given patient, the idea is to generate multiple imputed data-sets by using fully conditional specification (FCS) [26] given the BMI and the age of the patient. Then, we apply the SCORE2 risk calculator for every imputed data-sets to compute the predicted risks. In the method I2, we take the mean of these risks as a single point prediction of the true risk. In contrast, in the method I5 we take into account the uncertainties of the imputation by considering the empirical distribution of the different risks as a probabilistic prediction.

**Fig 7:**
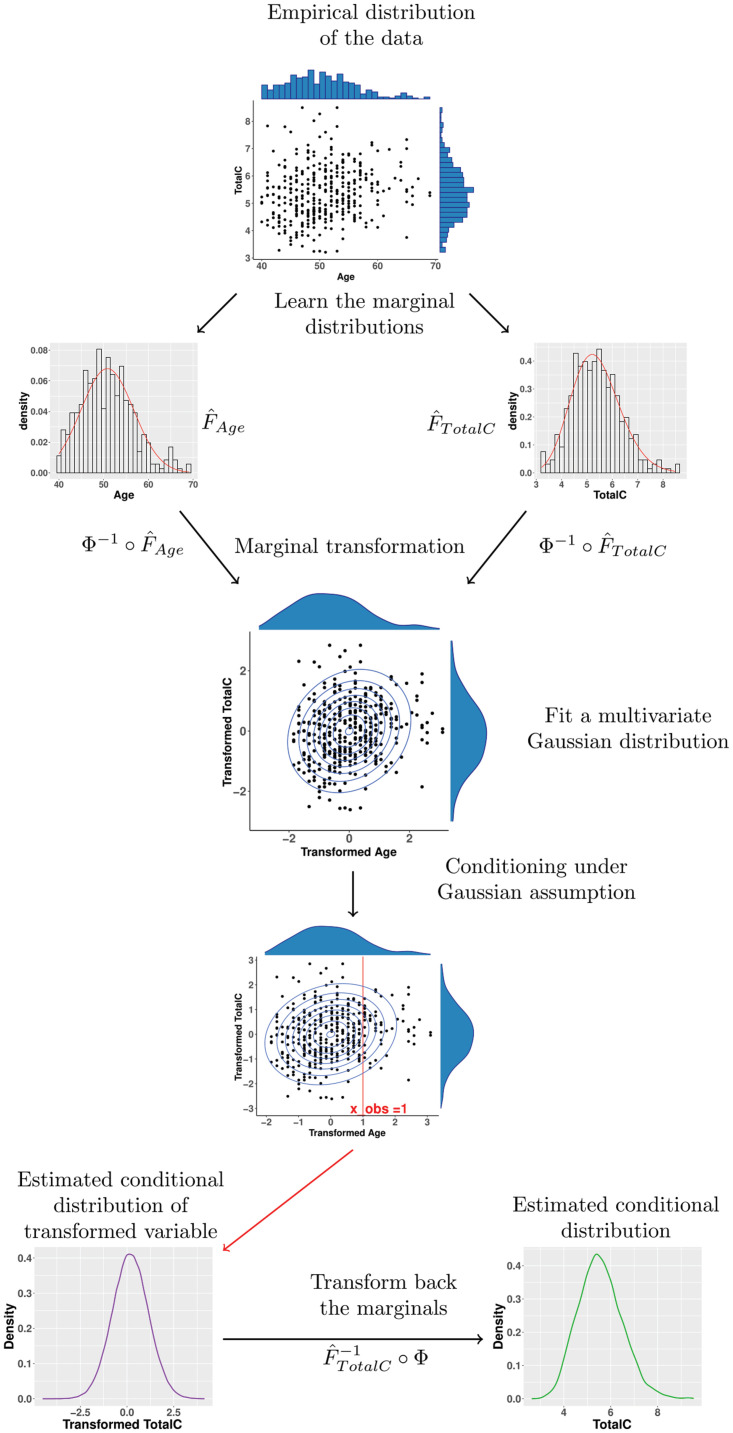
The scheme shows a simplified (univariate) version of the approach used in I3 and I4. Given the empirical distribution of (Age, TotalC) in form of a data set, we model the conditional distribution of TotalC given Age.

We now focus on the methods I3 and I4. The idea is to first estimate the conditional distribution of the *unknown inputs* (BP, TotalC, HDLC) given the *known inputs* (Age, BMI). In order to sample from the estimated conditional distribution of the unknown inputs given the known inputs, we use the approach graphically explained in Fig 7.

We can then plug the (sample) mean of this distribution together with the known inputs (including smoking habits and diabetes) into the model of the SCORE2 risk calculator and obtain a prediction of the risk (I3). The conditional risk distribution (I4) can similarly be approximated by sampling multiple values from the conditional distribution of (BP, TotalC, HDLC) given (Age, BMI), calculating the SCORE2 risk with the risk calculator for each sample (together with the known inputs) and collect the resulting values.

Let us remark that these approaches are very generic and could be applied with a range of options when it comes to recovering the marginal distributions as well as the involved dependence structure (copula). Here for simplicity we focus on an ad hoc approach that relies on a multivariate Gaussian dependence structure. The promising results on the modest data set covered here pave the way to further investigations on broader classes of problems. In particular, we expect that more elaborated copula methods may stand out when working with larger data sets.

We would like to stress that the univariate transformations to obtain Gaussian marginals would not generally ensure a Gaussian dependence structure (in fact whether or not the underlying copula is Gaussian would not be affected by strictly increasing marginal transformations such as considered here). This procedure rather corresponds to assuming that the vector of inputs stems from a multivariate Gaussian vector that has been marginally transformed and from which we would recover the marginal transformations and the underlying covariance structure empirically. Although this is somewhat an arbitrary model and was chosen for the convenience of its implementation, it turned out to work surprisingly well in this study and could not be significantly outperformed in terms of risk score estimation by our attempts to work with more complex dependence structures.

Table 1 shows the parameters of the estimated distributions for the marginals.

**Table 1:** Estimates for the marginal distributions with parameter values.

variable (abr.)	distribution family	parameters
age (Age)	Normal	(50.82, 5.88)
body mass index (BMI)	log-Normal	(3.22, 0.19)
total cholesterol (TotalC)	Gamma	(31.48, 0.17)^†^
hdl cholesterol (HDLC)	log-Normal	(0.54, 0.30)
systolic blood pressure (BP)	Gamma	(59.86, 2.07)^†^

See Figs 2–6 for Q-Q-plots.

^†^ Shape and scale parameter.

### Imputation examples with a selection of artificial patients

**Fig 8:**
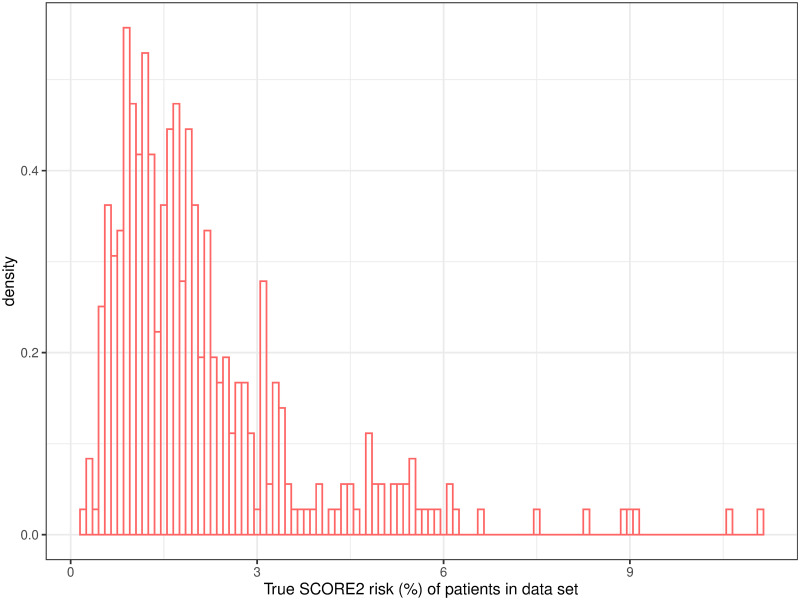
For each patient in the data set we can calculate the risk of cardiovascular diseases based on the SCORE2 algorithm. The above histogram shows these SCORE2 risks in relative numbers.

The SCORE2 risk prediction model uses the values age, smoking habit, systolic blood pressure, total cholesterol, HDL cholesterol and incidence of diabetes, that are all contained in the underlying data set. Hence we can calculate the cardiovascular risk based on this risk model for each individual in our data set and plot the distribution of the SCORE2 risk over our data set using a simple histogram, see Fig 8.

Assume now that we want to calculate the SCORE2 risk for cardiovascular disease of an additional individual that is not contained in the given data set. A quite straightforward and naive approach for an estimation of the SCORE2 risk of our additional individual would be to take the sample mean risk of the patients in the given data set (2.1%).

However, when considering the sample mean risk, we ignore completely that some things about our patient may be known. Therefore, we want to use the known information to obtain a better estimate. The known information here is age, smoking habit, incidence of diabetes and additionally the BMI for each individual. Hence, we can assume that these parameters of our additional individual are given. However, blood pressure, total cholesterol and HDL cholesterol can only be obtained through measurements that need to be performed by a practitioner and hence we assume these parameters to be unknown. In the following we will illustrate our approach using three artificial individuals with high, medium and low value of the (true) associated SCORE2 risk.

**Example 1**.

Consider a potential patient with the following parameter values.

**Table:** 

**Age**	**Smoker**	BP	TotalC	HDLC	**Diabetes**	**BMI**	True SCORE2 Risk
**60**	**1**	150	5.1	1.6	**0**	**23**	11.2

**Fig 9:**
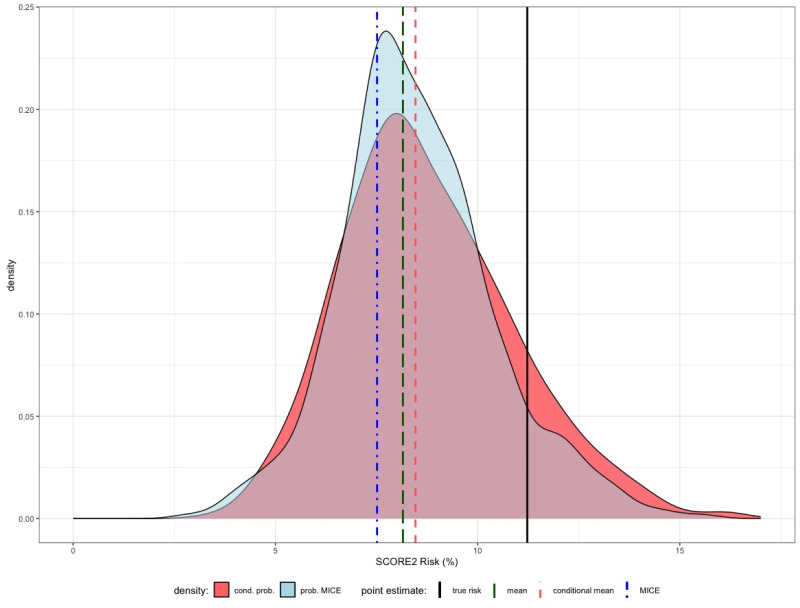
We assume that for the artificial patient, it is only known that she is a 60 years old woman, smoker, no incident of diabetes with a BMI of 23 kg/m^2^. The remaining inputs for the calculation of the SCORE2 risk are considered to be unknown and have to be estimated.

Let us assume now that we only know about this potential patient that she is a 60 years old woman, smoker, with no incidence of diabetes and a BMI of 23 kg/*m*^2^. The missing values to calculate the SCORE2 risk are then all clinical inputs (BP, TotalC and HDLC). In accordance with approach I1, we can now substitute these missing values with the sample mean blood pressure (124 mmHg), sample mean total cholesterol (5.4 mmol/L) and sample mean HDL cholesterol (1.8 mmol/L). The predicted SCORE2 risk (8.2%) is now much higher than our previous naive estimation and displayed by the dark green long-dashed line in Fig 9.

We now move to the more elaborated approaches and compare them to the popular Multiple Imputation by Chained Equations (MICE) method. The sample mean of the MICE approach is displayed by the dot-dashed blue line in Fig 9 with an estimate for the SCORE2 risk of 7.7%.

The approach I3 delivers again a point prediction, where we are using the conditional distribution of the unknown inputs given the known inputs of our individual. Instead of using the sample means for the unknown inputs we use the mean of the conditional distribution of the covariates (BP, TotalC, HDLC) given the known values (Age = 60, BMI = 23) of our patient. The conditional means are in this case

$$\begin{array}{c} \left({\text{BP}}_{\text{cond}},{\text{TotalC}}_{\text{cond}},{\text{HDLC}}_{\text{cond}}\right)=\left(126,5.6,1.8\right). \end{array}$$

Comparing the true unknown input values, we see that the estimated values for HDL and total cholesterol are slightly higher than the true HDL and total cholesterol, but the ratio HDLC/TotalC is almost the same in both cases. HDL cholesterol can be considered as the “good cholesterol” and a higher percentage of it in the total cholesterol tends to lower the SCORE2 risk, but its difference is too insignificant to account for the lower predicted risk. However, the blood pressure prediction seems to be far off. In Fig 9 this results in a too low conditional mean of 8.5% (red dashed line in Fig 9).

More generally, we can also use the full joint conditional distribution of the covariates (BP, TotalC, HDLC) given (Age = 60, BMI = 23) or a probabilistic MICE approach (red and blue graph in Fig 9) to account for the uncertainty that is not displayed with simple point estimates. Using *n* = 500 Monte Carlo samples, this yields for both approaches a distribution of the SCORE2 risk for our additional individual (probabilistic forecast). Notice that the true risk is still part of the red and blue graphs. This means that for some extreme sample values of the conditional distribution of (BP, TotalC, HDLC) given (Age = 60,BMI = 23) and some MICE iterations, we obtain an estimate for the SCORE2 risk that is close to the true risk.

**Example 2**.

Assume now that we have a different artificial individual with the following measurements.

**Table:** 

**Age**	**Smoker**	BP	TotalC	HDLC	**Diabetes**	**BMI**	True SCORE2 Risk
**65**	**0**	110	5.5	1	**0**	**37**	4.3

**Fig 10:**
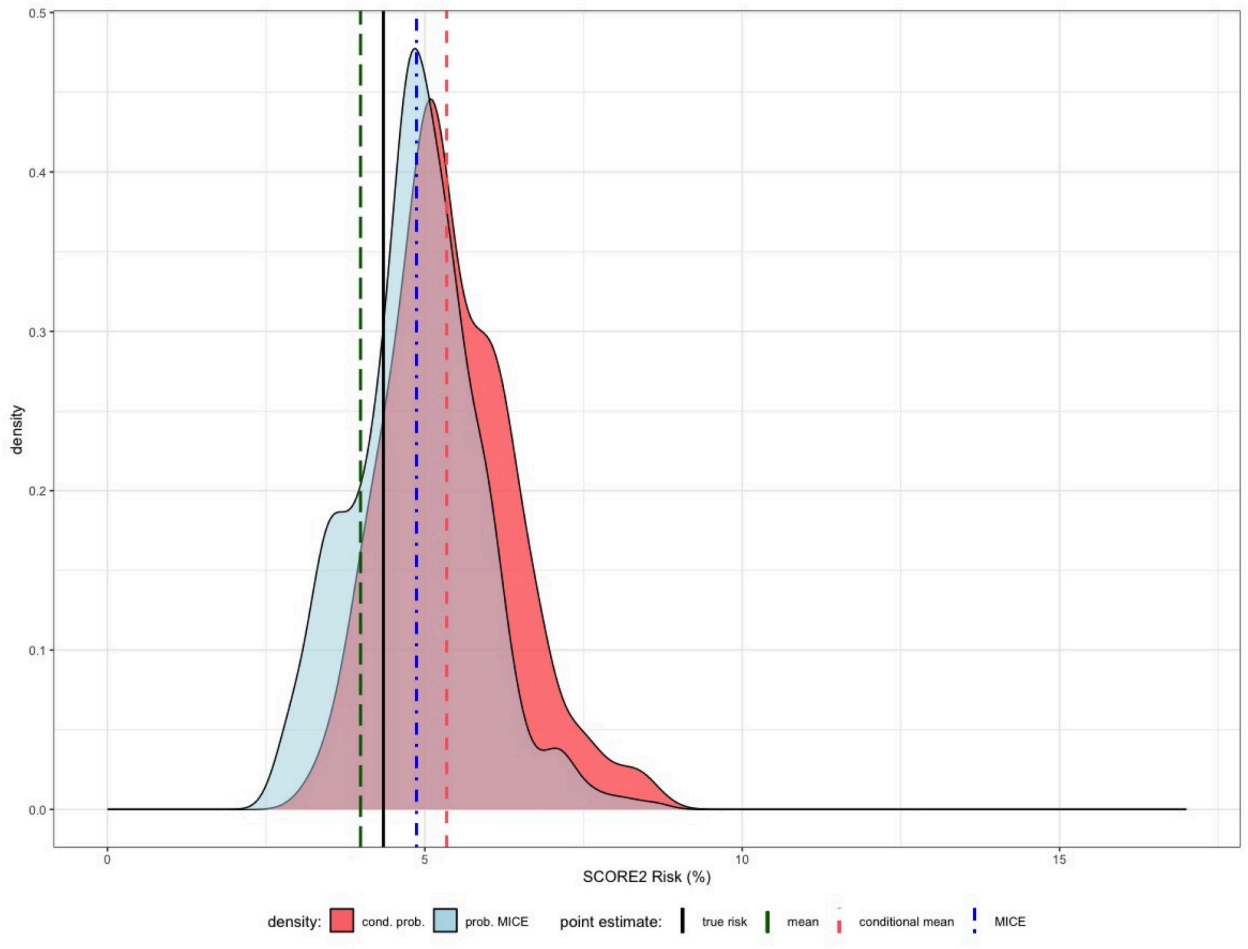
In this example we assume that for the artificial patient, it is only known that she is a 65 years old woman, non-smoker, no diabetes with a BMI of 37 kg/m^2^. The remaining inputs for the calculation of the SCORE2 risk are again considered to be unknown and have to be estimated.

The true SCORE2 risk of the patient is again displayed as vertical black line at 4.3% and the naive approach of taking the sample mean for the unknown inputs (green dashed line in Fig 10) gives an estimate for the SCORE2 risk of 4%.

We want again to use the mean of the conditional distribution of (BP, TotalC, HDLC) given the known values (Age = 65, BMI = 37) of our additional individual as inputs for the unknowns. The conditional means are here given by

$$\begin{array}{c} \left({\text{BP}}_{\text{cond}},{\text{TotalC}}_{\text{cond}},{\text{HDLC}}_{\text{cond}}\right)=\left(138,5.8,1.3\right) \end{array}$$

and yield finally a point estimate of the SCORE2 risk of 5.3%, which is slightly worse than the MICE estimate of 4.9%. Note that the estimates for systolic blood pressure, total cholesterol and HDL cholesterol above are indeed different from the ones we have obtained in Example 1 due to the slightly different values for Age and BMI. In the next example the impact will become even more clear. In Fig 10 we see again not only the point estimates (vertical lines) but also the conditional distribution of the SCORE2 risk given (Age = 65, BMI = 37) (red graph) and the probabilistic MICE (blue graph).

**Example 3**.

At last, we present an example where the true SCORE2 risk is rather low. Assume that we have an artificial patient with the following parameters.

**Table:** 

**Age**	**Smoker**	BP	TotalC	HDLC	**Diabetes**	**BMI**	True SCORE2 Risk
**50**	**0**	100	4	1.6	**0**	**22**	0.9

The conditional mean for the unknown inputs given the known values (Age = 50,BMI = 22) is here

$$\begin{array}{c} \left({\text{BP}}_{\text{cond}},{\text{TotalC}}_{\text{cond}},{\text{HDLC}}_{\text{cond}}\right)=\left(120,5.3,1.9\right). \end{array}$$

**Fig 11:**
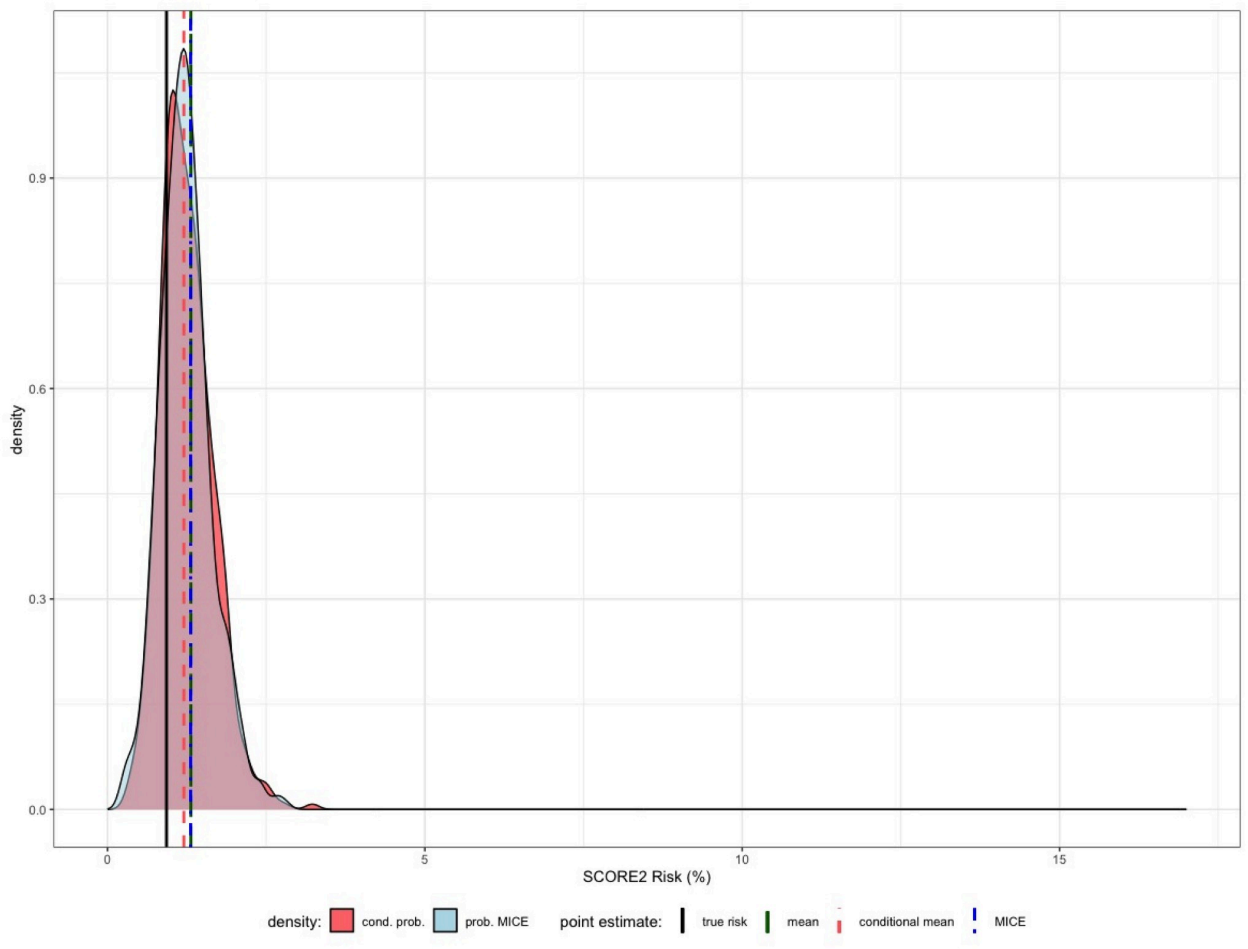
In this last example we assume that the artificial patient is a 50 years old woman, non-smoker, no diabetes with a BMI of 22 kg/m^2^. The remaining inputs for the calculation of the SCORE2 risk are again assumed to be unknown and have to be estimated.

Fig 11 shows the results. We can see that in comparison to our previous examples, the probabilistic approaches have much lower standard deviation. This can be observed for all individuals where the true SCORE2 risk tends to be small. Both observations are due to the characteristics of our data set, since the majority of the patients has a SCORE2 risk below 2%.

### Benchmark

Let us now move away from specific examples and examine our methods’ predictive ability by means of scoring rules. As a first scoring rule we consider the Continuous Ranked Probability Score (CRPS).

To assess the predictive ability of our methods, we tried to predict the SCORE2 risk for every patient in our data set. To this end, we used the leave-one-out cross-validation approach. In other words, the patient whose risk was supposed to be estimated was treated as the test set, while the remaining patients formed the training set used for the estimation. This procedure was repeated for every patient and for the following three scenarios: In the first scenario, only the total cholesterol level (TotalC) of the patient was assumed to be unknown and had to be estimated. In the second scenario, the lipid levels (TotalC and HDLC) were treated as unknown. This is indeed an important scenario, since the blood pressure can be easily measured at the pharmacy or even at home. Finally, in the third scenario, all clinical parameters (TotalC, HDLC and BP) were treated as unknown. Table 2 displays the average CRPS scores for all five methods and all four scenarios.

**Table 2:** Mean CRPS scores for I1-I5 depending on how many clinical parameters were unknown. The smallest score for each scenario is highlighted in bold.

Method	TotalC	TotalC, HDLC	TotalC, HDLC, BP
**I5**	0.101	0.257	**0.346**
**I4**	**0.100**	**0.256**	0.352
I3	0.142	0.355	0.483
I2	0.153	0.383	0.526
I1	0.145	0.395	0.547

In Table 2, the probabilistic prediction approaches alternately yield the smallest CRPS scores among the three scenarios. While in the simpler cases of one or two systematically missing values I4 slightly outperforms I5, I5 does clearly outperform I4 in the case where all clinical parameters are missing. A reasonable reservation to Table 2 is that the method where the conditional distribution is predicted needs a larger training set to obtain good predictions.

Another objection may be that the CRPS might not be an ideal choice of score as a wrong prediction of a low risk may have severe consequences, since in such a case the patient may then not feel the need to see a practitioner. In order to also put a specific focus on this situation, we further considered the Brier score for associated probabilities. In particular, we looked at the Brier score that measures a forecast’s ability to predict whether or not the SCORE2 risk is above 5%. Table 3 displays the results.

**Table 3:** Mean Brier scores for I1-I5 measuring the estimates’ ability to identify risks above 5% depending on how many clinical parameters were unknown. The smallest score for each scenario is highlighted in bold.

Method	TotalC	TotalC, HDLC	TotalC, HDLC, BP
**I5**	**0.008**	0.022	**0.031**
**I4**	**0.008**	**0.020**	0.033
I3	0.014	0.033	0.047
I2	0.011	0.036	0.039
I1	0.011	0.042	0.045

Again, I4 and I5 outperform the other approaches. While I4 and I5 yield the same Brier score for the scenario where only TotalC is missing, I4 has a lower Brier score when both blood lipid values are missing, and I5 has a lower Brier score when all clinical values are missing. For completeness, we also look at the Brier score that measures the forecasts’ ability to predict risks below 1%, to see whether some of the approaches tend to overestimate the risk. Table 4 displays the results. Both methods, I4 and I5, perform very similarly, when it comes to identify low risk patients.

**Table 4:** Mean Brier scores for I1-I5 measuring the estimates’ ability to identify risks below 1% for the four approaches depending on how many clinical parameters were unknown.

Method	TotalC	TotalC, HDLC	TotalC, HDLC, BP
**I5**	**0.027**	**0.072**	**0.089**
**I4**	0.028	**0.072**	0.090
I3	0.039	0.100	0.134
I2	0.05	0.109	0.128
I1	0.042	0.111	0.153

## Discussion

### Sensitivity analysis of the probabilistic approaches I4 and I5

The advantage of the probabilistic approaches I4 and I5 is clearly that they do not only provide a point prediction, i.e. a real-valued estimator for the SCORE2 risk of a patient, but a full distribution of the SCORE2 risk of this patient, that results from underlying uncertainties and reflects these regarding the unknown variables. However, in practical decision-making situations, it may be required to perform deterministic classifications based on the latter. Now, if we want to transform the probabilistic forecast into a concrete point estimate for the SCORE2 risk, there are many possibilities. One possibility is the already mentioned conditional mean (I3) that we have used in the previous examples and evaluations. Another approach would be to use the median or more general a particular quantile of the distribution.

**Fig 12:**
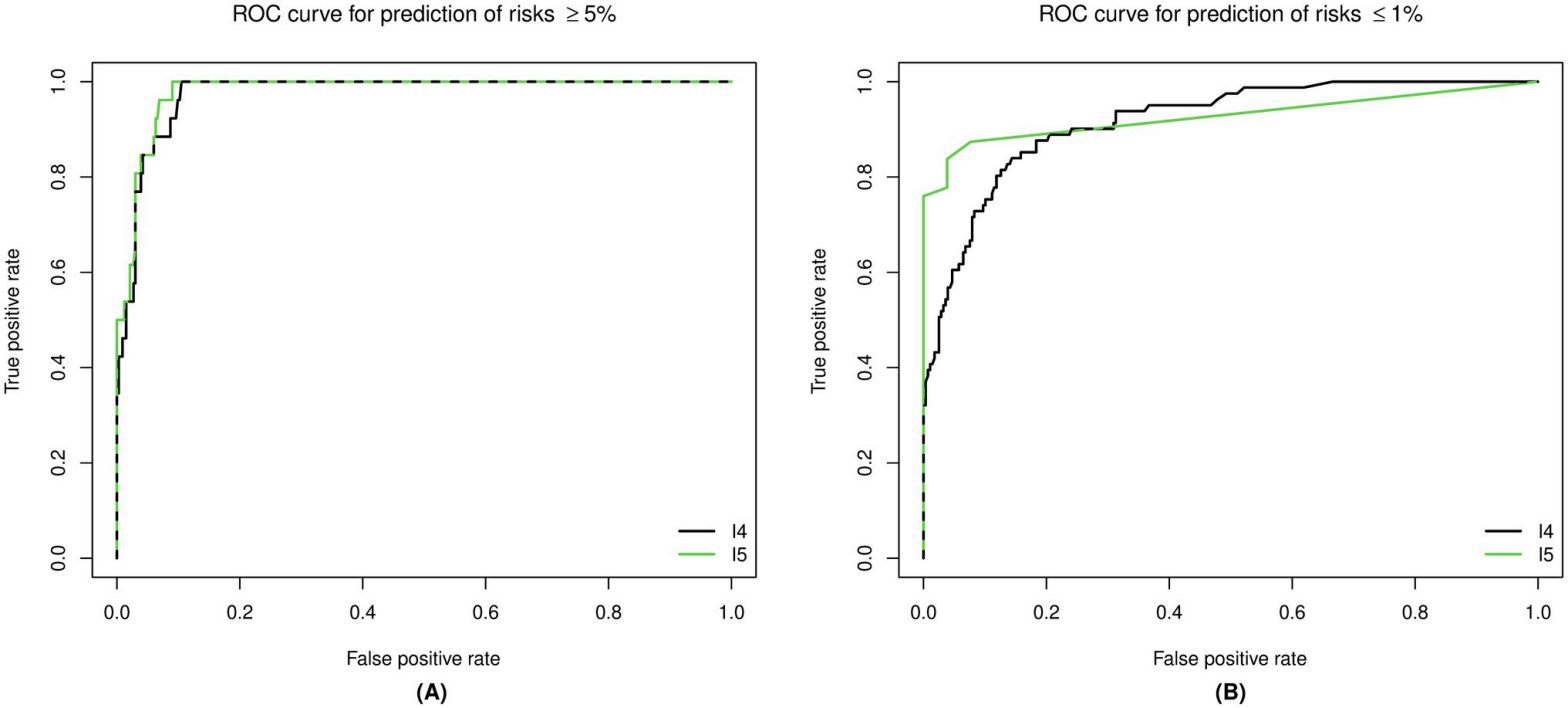
ROC curve for classification of patients with a risk higher than 5% (A) and patients with a risk lower than 1% (B). The green line represents the ROC curve of I5, and the black line corresponds to the ROC curve of I4. If both curves overlap the lines are dashed.

If a probabilistic recommendation should be made, i.e. to decide whether a patient is high risk (>5%) or low risk (<1%), the question arises if there exists ideal cut-off-probabilities *α*_*h*_, *α*_*l*_ ∈ [0, 1], in the sense that every patient with a probability of having a risk higher than 5% (or lower than 1%) above this threshold *α*_*h*_ (or *α*_*l*_) should be considered as a high risk patient (or low risk patient). This means we are checking if for each patient

$$\begin{array}{c} 1-I\;P\left(\text{SCORE2}\;\text{risk}\leq 5\%|X_{\text{known}},X\right)\geq \alpha_{h} \end{array}$$

for the high risk classification and similarly

$$\begin{array}{c} I\;P\left(\text{SCORE2}\;\text{risk}\leq 1\%|X_{\text{known}},X\right)\geq \alpha_{l}, \end{array}$$

for the low risk classification, where the left hand side denotes the conditional probability of the SCORE2 risk given the known variables of the patient ***X***_known_ and the underlying data ***X***. We can then calculate the false-positive and true-positive rate that results from this classification approach for different cut-off-probabilities *α*. The R-package ROCR automates these calculations and returns so-called ROC-curves (see Fig 12). The ROC-curves show how the false-positive and true-positive rates change for decreasing values of *α* ∈ [0, 1]. Note that the left end of both graphs corresponds to *α* = 1 and the right side to *α* = 0. The underlying *α* in-between is not explicitly displayed. The ROC curves of I4 and I5 show, that I5 slightly outperforms I4 when it comes to identifying high risk patients. When considering the task of identifying low risk patients, both approaches have different strengths and weaknesses. However, the ROC curve for I5 does have a smaller area under the curve.

The most important aspect of probabilistic recommendation is of course to correctly classify high risk patients. Since the resulting invasive procedures resulting from a (also possibly false) high risk classification are very small (blood sample), a rather small value for *α* would appear sensible here. For I4 the Table 5 displays the classification results for *α* ∈ {0.01, 0.05, 0.1, 0.3}. For I5 the results are displayed in Table 6. Looking at those tables, we see that the number of correctly predicted high risk patients decreased with increasing values of *α*. On the other hand, the number of correctly classified low risk patients increases with increasing values of *α*. However, when looking at our very high risk patient (Example 1) from Section Imputation examples with a selection of artificial patients, we see that this patient would be classified correctly for all *α* ≤ 0.95, since $1-I\;P(\text{SCORE2}\;\text{risk}\leq 5\%|X_{\text{known}},X)=0.95$ (percentile of the conditional distribution). Clearly, the data set is of moderate size and “very high risk” is to be understood in a relative sense, calling for larger scale studies when it comes to statistical analysis of rare events and extreme values.

**Table 5:** Classification for risk below and above 5% under a cut-off-probability of *α* for method I4.

	risk prediction below 5%	risk prediction above 5%
**α = 0.01**		
true risk below 5%	275	58
true risk above 5%	0	26
**α = 0.05**		
true risk below 5%	306	27
true risk above 5%	3	23
**α = 0.1**		
true risk below 5%	317	16
true risk above 5%	4	22
**α = 0.3**		
true risk below 5%	323	10
true risk above 5%	9	17

**Table 6:** Classification for risk below and above 5% under a cut-off-probability of *α* for method I5.

	risk prediction below 5%	risk prediction above 5%
**α = 0.01**		
true risk below 5%	277	56
true risk above 5%	0	26
**α = 0.05**		
true risk below 5%	308	25
true risk above 5%	1	25
**α = 0.1**		
true risk below 5%	318	15
true risk above 5%	4	22
**α = 0.3**		
true risk below 5%	324	9
true risk above 5%	10	16

It is also of interest to look at the classification tables for methods I1-I3. The results are shown in Table 7. As expected, more high risk patients are correctly classified using I2 and I3 than when using I1. While method I2 classifies slightly more high risk patients correctly, method I3 is slightly better in correctly classifying low risk patients. However, the probabilistic methods I4 and I5 clearly tend to be more sensitive and less specific.

**Table 7:** Confusion matrix for the point estimate methods I1-I3.

	risk prediction below 5%	risk prediction above 5%
**I3**		
true risk below 5%	329	4
true risk above 5%	13	13
**I2**		
true risk below 5%	331	2
true risk above 5%	12	14
**I1**		
true risk below 5%	333	0
true risk above 5%	16	10

### Accuracy of the imputations

To evaluate our imputation methods, we have compared the risk predictions obtained, by using respectively the imputed and the original data, after applying SCORE2 risk-calculator. This is an inference specific utility measure and is useful to compare different imputation methods for a given prediction problem. This is to be contrasted with settings where the focus is on the quality of imputation directly in terms of the similarity between the prediction of missing inputs and their actual values. As already mentioned before, copula approaches beyond the Gaussian case did not result in significant improvements in terms of SCORE2 predictions.

Nonetheless, by adopting more intricate dependence structures like vine copulas or Gaussian mixture copulas, we can achieve greater modeling flexibility. However, this comes with the challenge of more complex copula parameters estimation.

### Extra covariates

The SCORE2 risk relies on the covariates age, smoking behavior, systolic blood pressure, total cholesterol, HDL cholesterol and diabetes diagnosis of the patient. For the results presented in Section Benchmark, however, we additionally used the patient’s BMI as a covariate. This additional knowledge was assumed to be informative here, by helping refining predictions for lipid values (HDL cholesterol, total cholesterol). Naturally, given that the sample size is large enough for reasonable predictions, considering other additional covariates that strongly correlate with the unknown clinical parameters may improve predictions even more and reduce uncertainty.

Indeed, the association between the lipids and the BMI has been tested previously in different studies, i.e. it has been shown that total cholesterol and the ratio of total cholesterol to HDL cholesterol tends to increase with increasing BMI, while HDL cholesterol tends to decrease for increasing BMI [27–29]. Recently it has been shown that this correlation is still valid for postmenopausal women, where body composition changes with body fat accumulation, resulting in an increase in cardiometabolic risk factors [30]. This complements previous findings for pre-menopausal women [31]. Especially the beneficial impact of high HDL cholesterol levels on cardiovascular diseases has been pointed out [32].

The results confirmed the correlations that we discovered in our underlying dataset and motivated the inclusion of BMI as additional covariate. To illustrate that the inclusion of the BMI as an additional covariate did indeed improve our risk predictions, we reran our calculations and only considered the covariates that are directly necessary for calculating the SCORE2 risk. Table 8 displays the results.

**Table 8:** Mean scores for I1-I5 for the case where all clinical parameters are unknown. Compared to the tables in Section Benchmark instead of BMI and age only the age was used as an additional covariate for the prediction.

Method	$\bar{\text{CRPS}}\left(\hat{F},y\right)$	${\bar{\text{Brier}}}_{>5\%}\left(\hat{F},y\right)$	${\bar{\text{Brier}}}_{<1\%}\left(\hat{F},y\right)$
**I5**	**0.385**	**0.032**	**0.107**
**I4**	**0.385**	0.033	**0.107**
I3	0.529	0.047	0.153
I2	0.543	0.045	0.164
I1	0.547	0.045	0.153

The methods I2-I5 did indeed benefit from the additional covariate BMI, as the scores were smaller when the BMI was included in the estimation process. The scores of I1 are identical to Section Benchmark. This is not surprising, as the BMI did not play a role in this method.

### Sample size

**Fig 13:**
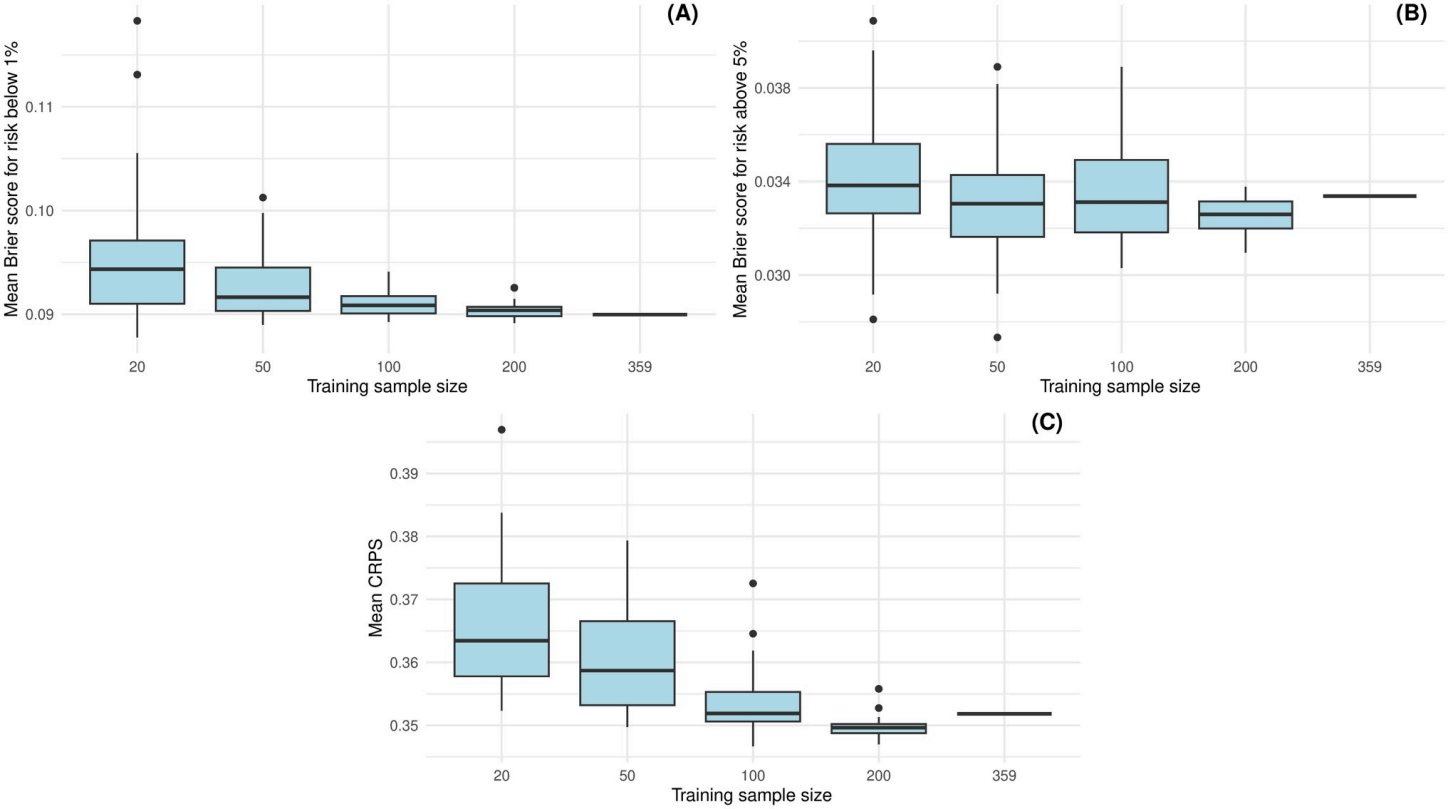
The boxplots show how approach I4 behaves when using smaller sample sizes (random subsets of the original data set). (A) refers to the Brier score for risk below 1%, (B) to the Brier score for risk above 5% and (C) to the CRPS.

The imputation approach can also be used to determine the necessary sample size for a follow-up study. To illustrate the procedure, we reran our calculations for differently sized subsets of the original data set and then studied their score. To avoid wrong conclusions due to a lucky or unlucky subset, we repeated this procedure twenty times. Fig 13 displays boxplots of the Brier scores for detecting risks below 1%, above 5% and the CRPS scores, respectively, using different sample sizes, i.e. random subsets of the original data set. As expected, decreasing the sample size increases the score variance. Looking at the boxplots in Fig 13, we suggest a sample size of at least 200 individuals to avoid decreasing the score too much.

## Conclusion

We compared several approaches to impute patient’s unknown parameters of HDL cholesterol, total cholesterol and blood pressure in the calculation of SCORE2 risks, relying on parameters known by the patient, i.e. age and BMI. We compared five different approaches by means of scoring rules. Our probabilistic prediction approaches I4 and I5 clearly outperformed the point prediction imputation approaches I1-I3. When compared in terms of CRPS (see Table 2) the probabilistic MICE approach I5 achieved better results when all three physician-recorded medical parameters are unknown, but was outperformed by I4 when only one or both lipid values are unknown. I5 achieved the best results for predicting high risk (Brier score high, SCORE2 risk >5%, see Table 4) and low risk patients (Brier score low, SCORE2 risk <1%, see Table 4), but in the latter case I4 and I5 are very close. This might be due to the small proportion of high risk patients in the underlying 359-patient data set. A possible improvement of approach I4, that should be investigated in further experiments, is to consider copula models beyond the Gaussian one. However, this should ideally be done using a data set with larger sample size.

Although the approach I4 is outperformed by I5 in the case where only the patient’s age is known, see Table 8, the comparison with Section Benchmark shows that especially I4 benefits from the use of additional patient data. An interesting perspective is the use of extra covariates such as provided, e.g., by wearable data, to try and reduce prediction uncertainty on those missing input variables with substantial effect.

The main advantage of the probabilistic approaches is shown in Section Sensitivity analysis of the probabilistic approaches I4 and I5 by the confusion matrices for classifying patients with a true risk above (or below) 5%. The ratios for the approaches I1 to I3 are fixed (see Table 7), whereas the ratio of the probabilistic approaches I4 and I5 depends on some cut-off parameter *α* ∈ (0, 1) that can be freely chosen (see Fig 12).
